# Influence of a montmorency cherry juice blend on indices of exercise-induced stress and upper respiratory tract symptoms following marathon running—a pilot investigation

**DOI:** 10.1186/s12970-015-0085-8

**Published:** 2015-05-11

**Authors:** Lygeri Dimitriou, Jessica A Hill, Ahmed Jehnali, Joe Dunbar, James Brouner, Malachy P. McHugh, Glyn Howatson

**Affiliations:** London Sport Institute, Middlesex University, Allianz Park, Greenland Way, NW4 1RLE London, UK; School of Sport, Health and Applied Science, St Mary’s University College, Twickenham, UK; Ipro Interactive Ltd, Oxfordshire, UK; School of Life Sciences, Kingston University, London, UK; Nicholas Institute of Sports Medicine and Athletic Trauma, Lenox Hill Hospital, New York, NY UK; Faculty of Health and Life Sciences, Northumbria University, Newcastle-upon-Tyne, UK; Water Research Group, School of Biological Sciences, North West University, Potchefstroom, South Africa

**Keywords:** Recovery, URTS, Exercise-induced inflammation, Muscle damage

## Abstract

**Background:**

Prolonged exercise, such as marathon running, has been associated with an increase in respiratory mucosal inflammation. The aim of this pilot study was to examine the effects of Montmorency cherry juice on markers of stress, immunity and inflammation following a Marathon.

**Methods:**

Twenty recreational Marathon runners consumed either cherry juice (CJ) or placebo (PL) before and after a Marathon race. Markers of mucosal immunity secretory immunoglobulin A (sIgA), immunoglobulin G (IgG), salivary cortisol, inflammation (CRP) and self-reported incidence and severity of upper respiratory tract symptoms (URTS) were measured before and following the race.

**Results:**

All variables except secretory IgA and IgG concentrations in saliva showed a significant time effect (*P* <0.01). Serum CRP showed a significant interaction and treatment effect (*P* < 0.01). The CRP increase at 24 and 48 h post-Marathon was lower (*P* < 0.01) in the CJ group compared to PL group. Mucosal immunity and salivary cortisol showed no interaction effect or treatment effect. The incidence and severity of URTS was significantly greater than baseline at 24 h and 48 h following the race in the PL group and was also greater than the CJ group (*P* < 0.05). No URTS were reported in the CJ group whereas 50 % of runners in the PL group reported URTS at 24 h and 48 h post-Marathon.

**Conclusions:**

This is the first study that provides encouraging evidence of the potential role of Montmorency cherries in reducing the development of URTS post-Marathon possibly caused by exercise-induced hyperventilation trauma, and/or other infectious and non-infectious factors.

## Background

Prolonged and exhaustive exercise is often associated with symptoms and signs of respiratory mucosal inflammation [1, 2]. The upper respiratory tract symptoms (URTS) usually seen following prolonged and exhaustive exercise [3, 4] have conventionally been attributed to a transient depression of the innate and adaptive immunity that eventually progresses into infection [5]. However, recent studies that examined the aetiology of URTS following Marathon running reported that half or more than two-thirds of symptomatic cases were attributable to inflammation [6] and/or allergy [7]. This non-infectious hypothesis can be further supported due to the fact, episodes of URTS in athletes are not characterised by usual seasonal patterns and show an unusual short-term duration [8]. Exercise-induced airway inflammation, common in endurance athletes [1, 9-11] can be mediated by a number of factors including the synergistic effect of hyperventilation trauma [2, 12], oxidative stress [13] and inhaled allergens and pollutants [7, 10, 14].

Exercise has shown to up-regulate the chemotactic cytokine expression in the airways [15] causing inflammation, allergic reactions in bronchi, increasing the likelihood of bronchoconstriction and possibly imitating symptoms that resemble respiratory infections [8]. For example, interleukin-8 (IL-8) has been implicated in pulmonary inflammation and hyper-responsiveness under acute oxidative stress [16, 17]. Previous studies have shown a unanimous increase in IL-8 following prolonged and exhaustive exercise [18, 19]. IL-8 is known to be a potent mediator of chemotaxis, and activates neutrophils resulting in the generation of reactive oxygen species (ROS) [20], which might lead to pulmonary inflammation and trauma [13]. Neutrophils increase markedly post-Marathon [19, 21], and pulmonary inflammation is characterised by the migration and activation of neutrophils into the airways [22]. Increased neutrophils in induced sputum post-Marathon have been reported in healthy athletes [1].

Tart Montmorency cherries are purported to be high in numerous phytochemicals, such as anthocyanins, and other polyphenolic compounds such as quercetin that possess anti-inflammatory and anti-oxidative properties [23, 24]. Growing interest in these functional foods has gained momentum in recent years and there is a mounting body of evidence to suggest Montmorency cherries can facilitate exercise recovery [24-28]; this is likely attributable to the increased bioavailability of these anti-inflammatory and anti-oxidative phytochemicals following ingestion [29, 30]. In a recent addition to the literature, Bell *et al.* [24] showed that in trained cyclists, consumption of a Montmorency cherry concentrate (in comparison to a calorific matched placebo) resulted in a reduction in lipid hyperoxides and a concomitant reduction in inflammation (IL-6 and C-reactive protein) following repeated days strenuous cycling. Additionally, polyphenols such as quercetin (also found in Montmorency cherries), modulate the expression of transcription nuclear factor-kappa B (NF-kappaB), [31, 32], which may in turn decreased the exercised-induced IL-6 production by an attenuation of cytokine transcription for IL-6. Previous studies have also shown these polyphenols to reduce other inflammatory biomarkers such as tumor necrosis factor alpha [32, 33], and macrophage inflammatory protein [33]. Consequently, it is conceivable that the anti-inflammatory and anti-oxidative potential of Montmorency cherries could attenuate the exercise-induced ‘stress’ response, immunity and URTS. Therefore, the aim of the current pilot study was to explore the possibility that Montmorency CJ supplementation before and following Marathon running could modulate markers of stress, immunity and self-reported upper respiratory tract symptoms.

## Methods

### Participants

Twenty Marathon runners (characteristics presented in Table 1) volunteered to participate. The subjects were the same cohort as those from previously published work [30] that examined the impact of Montmorency cherry juice blend on recovery following Marathon running. Eighteen completed the 2008 London Marathon (temp: 7 **°**C, humidity: 56 %, wind speed: 4 km/h) and remaining two completed the same distance in West London two weeks later in similar conditions (temperature: 7 **°**C, humidity: 50 %, wind speed: 12 km/h). Following completion of written informed consent, all participants were asked to refrain from taking nutritional supplements, pharmacological interventions and strenuous exercise (other than completing training runs prior to the Marathon) for the duration of the study. All procedures were granted ethical approval from the Institutional Research Ethics Committee, in accordance with the Helsinki Declaration.

**Table 1:** Study’s participant’s characteristics. No statistical differences found between groups for any variable; Values are mean ± SD

Group	Gender (M/F)	Age (years)	Stature (m)	Mass (kg)	Predicted time (h:min:ss)	Actual time (h:min:ss)	Highest weekly mileage	Longest training run (miles)	Past Marathons
**CJ**	7/3	37 ± 13	1.77 ± 0.06	72.9 ± 9.8	3:41:00 ± 0:26:01	3:48:04 ± 0:48:58	33.0 ± 11.6	20.9 ± 2.6	7 ± 9
**PL**	6/4	38 ± 5	1.75 ± 0.09	73.8 ± 9.5	3:56:40 ± 0:40:37	4:15:48 ± 1:01:22	31.7 ± 8.2	19.3 ± 3.1	2 ± 7

### Experimental overview

Participants were randomly assigned to either a placebo (PL) or cherry juice blend (CJ) group based upon predicted Marathon finish time. Possible sex differences in response to Marathon running were controlled by balancing the number of male and female participants in each group (3 CJ, 4 PL). Markers of stress, inflammation, mucosal immunity and upper respiratory tract symptoms were measured on four occasions; the day before the Marathon, immediately after, and at 24 h and 48 h after the Marathon. Following an initial visit to the laboratory, six days prior to the Marathon, participants were allocated to treatment groups and were instructed to take the supplement for five days prior to, the day of the Marathon and for the 48 h following the Marathon (total eight days).

### Treatment groups

The CJ group consumed 2 servings x 236 ml (taken morning and afternoon) of a fresh pressed blend (Cherrypharm Inc., Geneva, New York, USA) of tart Montmorency CJ combined with proprietary apple juice (which the manufacturers add to increase palatability). According to previous work [26, 28] each serving equated to 50-60 whole cherries and contained ~600 mg of phenolic compounds of which at least 40 mg were anthocyanins. The remaining compounds consisted of flavonoids such as quercetin, kaempferol and isoramnetin; flavanols such as catechin and epicatechin procyanidins and phenolic acids such as neochlorogenic acid, chlorogenic acid and ellagic acid. The estimated oxygen radical absorbance capacity (ORAC) value per serving was estimated as 55 mMol/L Trolox equivalents [26]. The PL group consumed 2 x 236 ml per day of a pre-made, sugar-free fruit flavored drink (Summer Fruits Squash, Tesco, UK) of similar appearance, but lacking the phytonutrient content and contained only a trace of anthocyanin.

### Incidence and severity of upper respiratory tract symptoms (URTS)

Runners were asked to report (adapted from Reid *et al.*, [34]) any incidence of cough; colored discharge; sore throat; watery eyes; nasal symptoms (congestion and/or discharge); sneezing and rate their severity on a 5-point Likert scale anchored by 1 (very mild) to 5 (very strong) as described by Nieman *et al.* [35]. Participants with two or more of the above symptoms present for a minimum of two consecutive days in the study period were identified as symptomatic [36].

### Saliva sampling procedures

Ten minutes before saliva collection, participants rinsed their mouths thoroughly for 30 s with water [37], and swallowed any saliva present in the mouth. Participants then actively swabbed their mouths, around their gums, tongue and inside their cheek, with an oral fluid collector (OFC; IPRO Interactive, Oxfordshire, UK) consisting of a synthetic polymer based material on a polypropylene tube, to collect saliva. The OFC has a volume adequacy indicator, giving a clear colour change when 1.0 mL (±20 %) is collected. Analyte recovery from the OFC is in excess of 85 % within 1 min of gentle shaking [38]. Saliva sample collection time was recorded (s), to facilitate the calculation of saliva flow rate (Sal_fr_), as described elsewhere [37], and was dependent on the time required by each individual to collect ~0.5 ml of saliva. The OFC was then inserted immediately in to an extraction buffer containing sodium phosphate, salts, detergents and preservatives designed to prevent growth of microorganisms and facilitate extraction of proteins and small mass molecular analytes from the swab. Samples were frozen immediately and stored at—20C until analysis [37].

### Salivary analyses

Secretory immunoglobulin A (sIgA), salivary immunoglobulin G (IgG) and salivary cortisol were determined in duplicate from the same sample, using enzyme immunoassay (EIA) test kits (IPRO Interactive Ltd., Oxfordshire, England), in an automated analyser (Tecan Nanoquant). The assay ranges were: sIgA 18.75–600 μg/mL; IgG 2.0–120 μg/mL; and cortisol 0.25–32.0 ng/mL. The intra-assay CV was: sIgA < 5.77 %; IgG < 3.37 %; cortisol < 7.85 %. The inter-assay CV was: sIgA < 12.52 %; IgG <10.77 %; and cortisol < 13.10 %. sIgA data is expressed as concentration (μg/mL) and as output/secretory rate (μg/min).

### Serum analyses

Serum C-reactive protein was determined using an automated analyser (c800, Abbott Architect). These data are published elsewhere [28], but are presented here as a global index of the exercise-induced inflammatory response. Normal ranges for this assay are <0.8 mg.L^-1^with minimum detection concentration (mdc) 0.3 mg.L^-1^. The CV of the intra-sample variability was 3.7 %. Samples with values below the mdc for any of the above markers were reported as equal to 0.5 mdc [39].

### Statistical analyses

Statistical analyses were performed using SPSS version 19.0. Values are reported as means and ± SD. An alpha level of 0.05 was chosen *a priori*. Independent T-tests were used to assess for demographic characteristics, predicted and actual Marathon time, Marathon history and training mileage leading up to the race between treatment groups. Differences between treatments were analysed using a 2 x 4 mixed model analyses of variance ANOVA with Treatment: CJ versus PL and Time as the within subject factor (pre, post, 24 h and 48 h). Mauchly’s sphericity test was used to assess if the variances of the differences between conditions were homogeneous. Simple main effects analyses were calculated for significant interaction effects between treatment and time. Violations of the sphericity assumption were corrected using the Greenhouse-Geisser estimate.

## Results

There were no differences between groups for age, stature, mass, previous Marathon history, weekly mileage, longest single training run, predicted and actual Marathon time (Table 1). Post-race body mass was lower than body mass the day before the race (P < 0.001) with similar declines in the CJ group and PL group (1.2 ± 1.3 kg *vs.* 1.7 ± 1.5 kg, respectively).

**Fig. 1:**
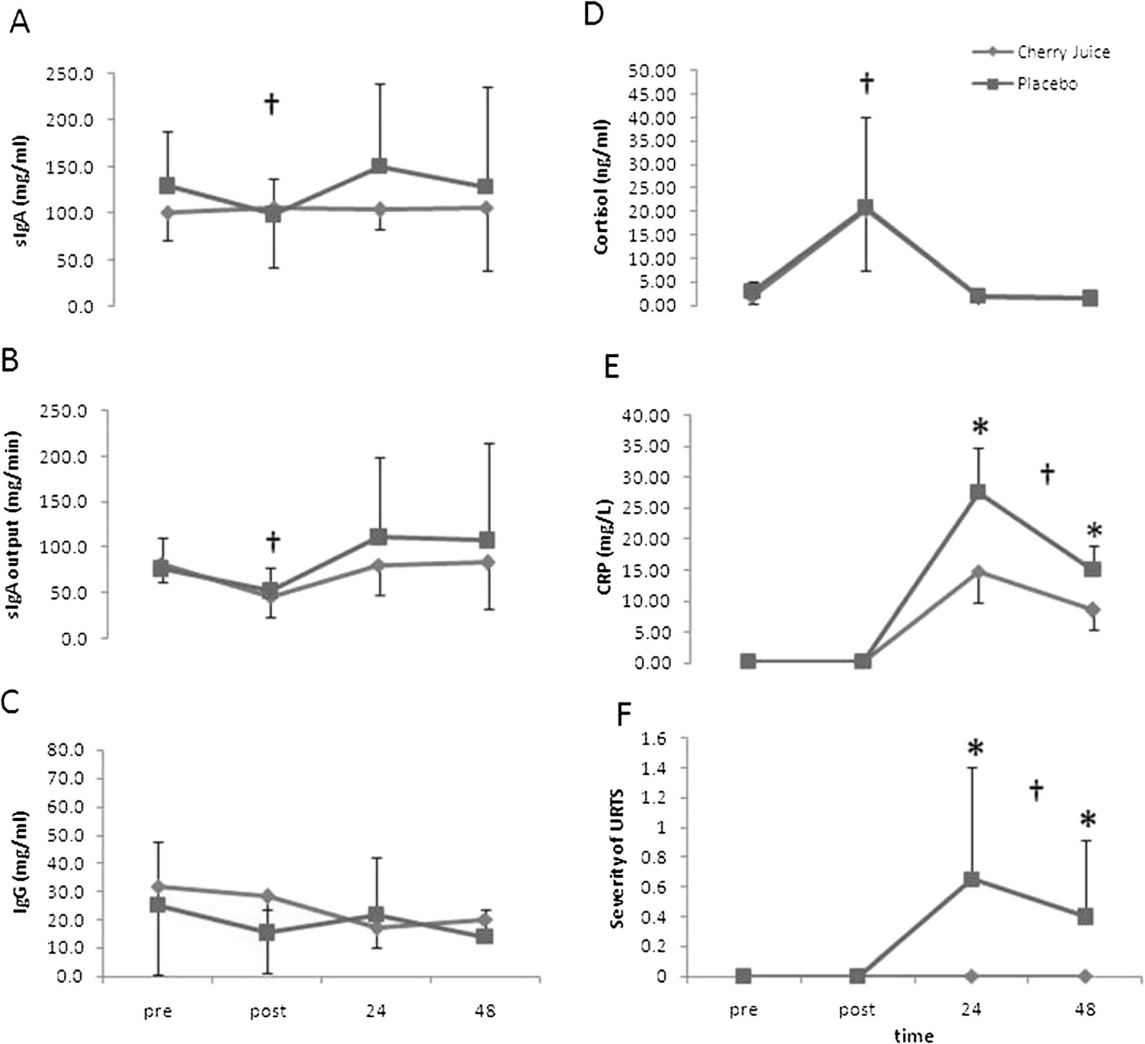
Selected markers of mucosal immunity, stress, inflammation and upper respiratory symptoms for the cherry juice and placebo groups before and up to 48 h following a Marathon race (Mean ± SD; n = 10 per group). sIgA concentration (panel **A**); sIgA output (panel **B**); IgG concentration (panel **C**); salivary cortisol (panel **D**); serum C-reactive protein concentration (CRP, panel **E**); severity of upper respiratory tract symptoms (URTS, panel **F**). *Significantly lower serum CRP and severity of URTS in the CJ than the PL group at 24 h and 48 h post-Marathon race (*P* < 0.05). †Significant time effect

Secretory IgA concentration showed no time or interaction effects (Fig. 1A). Conversely, there was a time effect for output (F(_3,54_) = 7.560, *P* < 0.001, η_p_^2^ = 0.296) and decreased immediately post-race in both groups when compared to pre-race levels, and returned to baseline by 24 h post-race. No treatment or interaction effects (*P > 0.05*) for sIgA were shown (Fig. 1B). Salivary IgG concentration showed no time, treatment or interaction effects (Fig. 1C).

Salivary Cortisol showed a significant time effect and was elevated immediately post-race in both groups, (F_(1,18)_ = 26.291, P < 0.001, η_p_^2^ = 0.594) when compared to pre-race levels, and returned to baseline by 24 h post-race. No significant treatment or treatment by time interaction effects were observed (see Fig. 1D).

Serum CRP showed a significant time effect (F(_3,54_) = 247.138, P < 0.001, η_p_^2^ = 0.932), a treatment by time interaction effect (F(_3,54_) = 10.667, P < 0.01, η_p_^2^ = 0.372), and a treatment effect F(_1,18_) = 12.920, P < 0.01, η_p_^2^ = 0.418). The increase in CRP at 24 and 48 h post-Marathon was significantly lower (F(_1,18_) = 12.14, P < 0.01 and F(_1.18_) = 9.88, P < 0.01, respectively) in the CJ group compared to PL group (see Fig. 1E).

The incidence and severity of URTS showed a time effect (F(_3,54_) = 6.359, P < 0.01, η_p_^2^ = 0.261). URTS were increased at 24 h and 48 h following the race when compared to pre-race levels in the PL group only. A treatment (F(1,18) = 7.826, P < 0.05, η_p_^2^ = 0.303) and interaction effect (F(_3,54_) = 6.359, P < 0.01, η_p_^2^ = 0.261) was observed, whereby URTS were significantly higher in the PL group at 24 h (F(_1,18_) = 7.57, P < 0.05) and 48 h post-race (F(_1,18_) = 5.44, P < 0.05) compared to CJ group (Fig. 1F). No URTS were reported in the CJ group at 24 h and 48 h post-Marathon as opposed to the PL group whom 50 % (5/10) of the runners developed URTS.

## Discussion

In this pilot study, we investigated the effects of a Montmorency tart cherry juice on markers of stress, immunity, and self-reported incidence and severity of upper respiratory tract symptoms following a Marathon. It was hypothesised that CJ supplementation would attenuate the cortisol and inflammatory response, reduce transient suppression of mucosal immunity and lower the development of URTS by protecting the respiratory tract from symptoms associated with infectious and non-infectious inflammatory agents following a Marathon. Despite no apparent change in cortisol or mucosal immunity between groups, runners that consumed Montmorency CJ had a lower CRP response at 24 and 48 h post–Marathon and had zero incidence of reported URTS up to 48 h after the Marathon, suggesting that CJ attenuated the exercise-induced inflammatory response and the subsequent development of URTS compared to the PL group following the race.

The development of URTS observed at 24 and 48 h post-Marathon in the PL group only, might be of a non-infectious nature reflecting a synergistic effect of pulmonary inflammation mediated by exercise-induced hyperventilation trauma [2, 12], oxidative stress [13], allergies [7] and air pollution [14]. A limitation with the current study is that we did not examine the prevalence of URTS beyond 48 h and this could be explored in future work given that URTS might become evident well beyond 48 h. Enhanced airway exposure to inhaled pollutants and/or allergens has been associated with airway hyper-responsiveness in many athletes of different sports [10]. Hyperventilation can cause bronchial dehydration injuries, excessive mucus production and/or airway oedema [40]; symptoms that could resemble an URTI. Airway inflammation is commonly reported in endurance athletes [1, 9-11], and heavy exercise is associated with pulmonary mucosal inflammation [2] induced by repetitive hyperventilation, bronchial dehydration [40], and increased airway osmolarity. Speculatively, these in turn might stimulate the release of chemotactic factors from bronchial epithelial cells [15], further supporting an exercise-induced URTS development attributable to non-infective inflammatory factors [41] in this study. The URTS usually seen following prolonged and exhaustive exercise [3] have conventionally been attributed to a transient immune depression that eventually progresses into infection [5]. However, recent studies that examined the aetiology of URTS following Marathon running reported that equal or more than two-thirds of symptomatic cases were attributable to inflammation [6] and/or allergy [7]. In the present study, CRP was significantly elevated in both groups but its response at 24 and 48 h following the Marathon was blunted in the CJ group compared to the PL. This is consistent with previous studies that used cherry supplementation [24, 28, 42, 43]. Furthermore, the CJ group did not report any URTS at 24 and 48 h post-Marathon as opposed to the PL group that reported a 50 % development of URTS. Further studies are needed to explore this in larger samples using techniques such as endobronchial biopsies and induced bronchoalveolar lavage fluid (BALF) to elucidate this possibility.

Findings from a previously published study that used the same cohort [28] showed a blunted IL-6 response immediately post-Marathon, and lower uric acid immediately and 24 h post-Marathon in the CJ group compared to the PL group. The bioactive food components (BAFC) contained in cherries have shown, *in vitro*, to inhibit cyclooxygenase (COX)-1 and COX-2 enzyme activity, by an average of 28 % and 47 % respectively, which is responsible for the inflammatory response [44]. A subsequent study further supported this by showing a COX-2 inhibitory effect of anthocyanins [45]. These aforementioned studies and the results from the cohort of this study suggest that the anti-inflammatory activities in tart cherries could attenuate the exercise-induced inflammatory response, its exacerbation and therefore the development of URTS. This observation adds to the growing body of evidence that shows the potential of tart Montmorency cherries in aiding exercise recovery and improving health indices [23, 24, 30, 32, 46, 47].

The prevalence of allergy and atopy (sensitization to common inhalant allergens) in long-distance runners has been reported to be 40 % and 49 %, respectively [7, 10]. In allergic diseases associated with the respiratory tract (*i.e.*, asthma and rhinitis) the migration of eosinophils to the mucosal surfaces is enhanced [48]. The respiratory tract is rich in cytokines and chemokines (*e.g.*, IL-8), which in turn could activate the eosinophils and possibly participate in the modulation of the local immune response via degranulation [49]. Eosinophil activation has been reported to be a crucial element in upper and lower respiratory inflammation [50]. Exercise-induced recruitment and degranulation of eosinophils and basophils to the respiratory tract due to airway inflammation may possibly explain the exercise-induced URTS development seen in the PL group in this study and the increased incidence of URTI reported previously [3]. Furthermore, a more recent *in vitro* study showed that the polyphenol quercetin suppresses eosinophil activation [51], suggesting that various BAFC of cherries might modulate eosinophil-mediated diseases, such as allergic rhinitis and asthma, which are very common pathologies in athletes. This idea is further supported by a study that showed that 58 % of runners with reported URTS following the 2010 London Marathon had allergy, as defined by a positive Allergy Questionnaire for Athletes (AQUA) and specific immunoglobulin E (IgE) response to various inhalant allergens [7]. The prevalence of the reported URTS following the 2008 London Marathon in this study were considerably lower (25 %) than previously reported (47 %) by Robson-Ansley *et al.* [7]. This difference could be partly explained by the daily average tree pollen counts on the day of the 2008 London Marathon that were approximately 9-fold less (Robson-Ansley *et al.* [7]) than those reported in the 2010 Marathon. Although this suggests a dose-dependent response between pollen counts and URTS, the antioxidant and anti-inflammatory properties of CJ offers a plausible explanation for the complete absence of reported URTS in the CJ group.

Pulmonary inflammation can be exacerbated by concurrent exposure to tropospheric ozone and other pollutants present in Metropolitan cities [14]. Exposure to air pollutants is greater in endurance athletes as they train mostly outdoors, and compete in Marathons that commonly take place in big cities. Furthermore, the shifting from nasal to oronasal or oral breathing leads to a greater inhalation of airborne allergens, pollutants, antigens and untreated air [7]. Pollutants increase the susceptibility to bacterial respiratory infections [14]. However, CJ ingestion could reduce exercise-induced URTI susceptibility since there are several cell culture studies that show the polyphenol quercetin to exert antipathogenic activities against a wide variety of viruses and bacteria, and to reduce infectivity of target cells and virus replication [52]. The results of the present study showed an absence of reported URTS development in the CJ group and might signify a down-regulation of the inflammatory pathways involved in pollutant inhalation. Furthermore, we cannot rule out that the absence of reported URTS in the CJ group might indicate an enhanced anti-pathogenic activity compared to PL. Future studies could investigate the interaction effect between air pollution and prolonged exhaustive exercise on the incidence of respiratory symptoms and bronchoconstriction, and identify possible prophylactic measures against them.

## Conclusions

The results of this pilot study showed that a Montmorency cherry juice blend appears to protect the URT from inflammatory symptoms caused by infectious and non-infectious agents, by possibly reducing the exercise-induced pulmonary inflammation. Modulation of the exercise-induced pulmonary inflammation by natural plant products might represent an attractive strategy to protect or alleviate the URT from inflammatory symptoms. This pilot investigation is the first to demonstrate preliminary evidence of the potential role of Montmorency cherry juice in reducing the development of URTS following long duration endurance exercise. Considering the limited sample size and healthy state of this study’s cohort, further studies with a larger sample size and participants with asthma, atopy, allergic rhinitis, exercise induced bronchoconstriction, airway hyper-responsiveness, and other pulmonary pathologies could be performed to explore the potential of cherries and other functional foods that might exert a similar effect.

## Abbreviations

AQUA: Allergy questionnaire for athletes
BAFC: Bioactive food components
CJ: Cherry juice
COX: Cyclooxygenase
EIA: Enzyme immunoassay
IgE: Immunoglobulin E
IgG: Immunoglobulin G
IL-6: Interleukin-6
IL-8: Interleukin-8
OFC: Oral fluid collector
PL: Placebo
ROS: Reactive oxygen species
Sal_fr_: Saliva flow rate
sIgA: Secretory immunoglobulin A
URTS: Upper respiratory tract symptoms

## References

[1] Bonsignore MR, Morici G, Riccobono L, Insalaco G, Bonanno A, Profita M (2001). Airway inflammation in nonasthmatic amateur runners. American J Physiol.

[2] Helenius I, Lumme A, Haahtela T (2005). Asthma, airway inflammation and treatment in elite athletes. Sports Med.

[3] Peters EM, Bateman E (1983). Ultramarathon running and upper respiratory tract infections. S Afr Med J.

[4] Nieman D, Johanssen L, Lee J, Arabatzis K (1990). Infectious episodes in runners before and after the Los Angeles Marathon. J Sports Med Phys Fitness.

[5] Nieman DC (1994). Exercise, upper respiratory tract infection, and the immune system. Med Sci Sports Exerc.

[6] Spence L, Brown WJ, Pyne DB, Nissen MD, Sloots TP, McCormack JG (2007). Incidence, etiology, and symptomatology of upper respiratory illness in elite athletes. Med Sci Sports Exerc.

[7] Robson-Ansley P, Howatson G, Tallent J, Mitcheson K, Walshe I, Toms C (2012). Prevalence of allergy and upper respiratory tract symptoms in runners of the London marathon. Med Sci Sports Exerc.

[8] Kuchar E, Miskiewicz K, Nitsch-Osuch A, Kurpas D, Han S, Szenborn L (2013). Immunopathology of exercise-induced bronchoconstriction in athletes—A new modified inflammatory hypothesis. Respir Physiol Neurobiol.

[9] Verges S, Devouassoux G, Flore P, Rossini E, Fior-Gozlan M, Levy P (2005). Bronchial hyperresponsiveness, airway inflammation, and airflow limitation in endurance athletes. CHEST J.

[10] Helenius I, Rytilä P, Metso T, Haahtela T, Venge P, Tikkanen H (1998). Respiratory symptoms, bronchial responsiveness, and cellular characteristics of induced sputum in elite swimmers. Allergy.

[11] Karjalainen E-M, Laitinen A, Sue-Chu M, Altraja A, Bjermer L, Laitinen LA (2000). Evidence of airway inflammation and remodeling in ski athletes with and without bronchial hyperresponsiveness to methacholine. Am J Respir Crit Care Med.

[12] Bermon S (2007). Airway inflammation and upper respiratory tract infection in athletes: is there a link. Exerc Immunol Rev.

[13] Guo R-F, Ward PA (2002). Mediators and regulation of neutrophil accumulation in inflammatory responses in lung: insights from the IgG immune complex model < sup > 1, 2</sup>. Free Radical Biol Med.

[14] Spannhake EW, Reddy SP, Jacoby DB, Yu X-Y, Saatian B, Tian J (2002). Synergism between rhinovirus infection and oxidant pollutant exposure enhances airway epithelial cell cytokine production. Environ Health Perspect.

[15] Bonsignore M, Morici G, Vignola A, Riccobono L, Bonanno A, Profita M (2003). Increased airway inflammatory cells in endurance athletes: what do they mean?. Clin Experimental Allergy.

[16] Ayyagari VN, Januszkiewicz A, Nath J (2004). Pro-inflammatory responses of human bronchial epithelial cells to acute nitrogen dioxide exposure. Toxicology.

[17] Epstein FH, Luster AD (1998). Chemokines—chemotactic cytokines that mediate inflammation. New England J Med.

[18] Ostrowski K, Rohde T, Asp S, Schjerling P, Klarlund Pedersen B (2001). Chemokines are elevated in plasma after strenuous exercise in humans. Eur J Appl Physiol.

[19] Suzuki K, Nakaji S, Yamada M, Liu Q, Kurakake S, Okamura N (2003). Impact of a competitive marathon race on systemic cytokine and neutrophil responses. Med Sci Sports Exerc.

[20] Gregory H, Young J, Schröder J-M, Mrowietz U, Christophers E (1988). Structure determination of a human lymphocyte derived neutrophil activating peptide (LYNAP). Biochem Biophys Res Commun.

[21] Niess AM, Sommer M, Schlotz E, Northoff H, Dickhuth H-H, Fehrenbach E (2000). Expression of the inducible nitric oxide synthase (iNOS) in human leukocytes: responses to running exercise. Med Sci Sports Exerc.

[22] Knaapen AM, Güngör N, Schins RP, Borm PJ, Van Schooten FJ (2006). Neutrophils and respiratory tract DNA damage and mutagenesis: a review. Mutagenesis.

[23] McCune LM, Kubota C, Stendell-Hollis NR, Thomson CA (2010). Cherries and health: a review. Crit Rev Food Sci Nutr.

[24] Bell PG, Walshe IH, Davison GW, Stevenson E, Howatson G (2014). Montmorency Cherries reduce the oxidative stress and inflammatory responses to repeated days high-intensity stochastic cycling. Nutrients.

[25] Kuehl KS, Perrier ET, Elliot DL, Chesnutt JC (2010). Research article Efficacy of tart cherry juice in reducing muscle pain during running: a randomized controlled trial.

[26] Connolly D, McHugh M, Padilla-Zakour O (2006). Efficacy of a tart cherry juice blend in preventing the symptoms of muscle damage. Br J Sports Med.

[27] Sumners D, Dyer A, Fox P, Mileva K, Bowtell J (2011). Montmorency cherry juice reduces muscle damage caused by intensive strength exercise. Med Sci Sports Exerc.

[28] Howatson G, McHugh M, Hill J, Brouner J, Jewell A, Van Someren KA (2010). Influence of tart cherry juice on indices of recovery following marathon running. Scand J Med Sci Sports.

[29] Bell PG, Gaze DC, Davison GW, George TW, Scotter MJ, Howatson G (2014). Montmorency tart cherry (< i > Prunus cerasus L.</i>) concentrate lowers uric acid, independent of plasma cyanidin-3-O-glucosiderutinoside. J Functional Foods.

[30] Bell P, McHugh M, Stevenson E, Howatson G (2014). The role of cherries in exercise and health. Scand J Med Sci Sports.

[31] Chen J-C, Ho F-M, Pei-Dawn Lee C, Chen C-P, Jeng K-CG, Hsu H-B (2005). Inhibition of iNOS gene expression by quercetin is mediated by the inhibition of IκB kinase, nuclear factor-kappa B and STAT1, and depends on heme oxygenase-1 induction in mouse BV-2 microglia. Eur J Pharmacol.

[32] Nair MP, Mahajan S, Reynolds JL, Aalinkeel R, Nair H, Schwartz SA (2006). The flavonoid quercetin inhibits proinflammatory cytokine (tumor necrosis factor alpha) gene expression in normal peripheral blood mononuclear cells via modulation of the NF-κβ system. Clin Vaccine Immunol.

[33] Comalada M, Ballester I, Bailón E, Sierra S, Xaus J, Gálvez J (2006). Inhibition of pro-inflammatory markers in primary bone marrow-derived mouse macrophages by naturally occurring flavonoids: analysis of the structure–activity relationship. Biochem Pharmacol.

[34] Reid V, Gleeson M, Williams N, Clancy R (2004). Clinical investigation of athletes with persistent fatigue and/or recurrent infections. Br J Sports Med.

[35] Nieman DC, Nehlsen-Cannarella SL, Fagoaga OR, Henson DA, Shannon M, Hjertman JM (2000). Immune function in female elite rowers and non-athletes. Br J Sports Med.

[36] Nieman DC, Henson DA, Gross SJ, Jenkins DP, Davis JM, Murphy EA (2007). Quercetin reduces illness but not immune perturbations after intensive exercise. Med Sci Sports Exerc.

[37] Dimitriou L, Sharp N, Doherty M (2002). Circadian effects on the acute responses of salivary cortisol and IgA in well trained swimmers. Br J Sports Med.

[38] Jehanli A, Dunbar J (2011). Skelhorn S, editors.

[39] Minetto M, Rainoldi A, Gazzoni M, Terzolo M, Borrione P, Termine A (2005). Differential responses of serum and salivary interleukin-6 to acute strenuous exercise. Eur J Appl Physiol.

[40] Anderson SD, Holzer K (2000). Exercise-induced asthma: Is it the right diagnosis in elite athletes?. J Allergy Clin Immun.

[41] Walsh NP, Gleeson M, Shephard RJ, Gleeson M, Woods JA, Bishop N (2011). Position statement part one: immune function and exercise.

[42] Jacob RA, Spinozzi GM, Simon VA, Kelley DS, Prior RL, Hess-Pierce B (2003). Consumption of cherries lowers plasma urate in healthy women. J Nutr.

[43] Kelley DS, Rasooly R, Jacob RA, Kader AA, Mackey BE (2006). Consumption of Bing sweet cherries lowers circulating concentrations of inflammation markers in healthy men and women. J Nutr.

[44] Seeram N, Momin R, Nair M, Bourquin L (2001). Cyclooxygenase inhibitory and antioxidant cyanidin glycosides in cherries and berries. Phytomedicine.

[45] Hou D-X, Yanagita T, Uto T, Masuzaki S, Fujii M (2005). Anthocyanidins inhibit cyclooxygenase-2 expression in LPS-evoked macrophages: Structure–activity relationship and molecular mechanisms involved. Biochem Pharmacol.

[46] Seeram NP, Aviram M, Zhang Y, Henning SM, Feng L, Dreher M (2008). Comparison of antioxidant potency of commonly consumed polyphenol-rich beverages in the United States. J Agric Food Chem.

[47] Howatson G, Bell PG, Tallent J, Middleton B, McHugh MP, Ellis J (2012). Effect of tart cherry juice (Prunus cerasus) on melatonin levels and enhanced sleep quality. Eur J Nutr.

[48] Djukanović R, Roche W, Wilson J, Beasley C, Twentyman O, Howarth P (1990). Mucosal inflammation in asthma. American Rev Respiratory Dis.

[49] MacKenzie JR, Mattes J, Dent LA, Foster PS (2001). Eosinophils promote allergic disease of the lung by regulating CD4+ Th2 lymphocyte function. J Immun.

[50] Choi GS, Kim JH, Shin YS, Ye YM, Kim SH, Park HS (2013). Eosinophil activation and novel mediators in the aspirin‐induced nasal response in AERD. Clin Experimental Allergy.

[51] Sakai-Kashiwabara M, Asano K. Inhibitory Action of Quercetin on Eosinophil Activation *In Vitro*. Evidence-Based Complementary Alternative Med. 2013;2013. 10.1155/2013/127105 PMC3690238 23840245

[52] Cushnie T, Lamb AJ (2005). Antimicrobial activity of flavonoids. Int J Antimicrob Agents.

